# Erbium-Doped Amorphous Carbon-Based Thin Films: A Photonic Material Prepared by Low-Temperature RF-PEMOCVD

**DOI:** 10.3390/ma7031539

**Published:** 2014-02-27

**Authors:** Hui-Lin Hsu, Keith R. Leong, I-Ju Teng, Michael Halamicek, Jenh-Yih Juang, Sheng-Rui Jian, Li Qian, Nazir P. Kherani

**Affiliations:** 1 Department of Electrical and Computer Engineering, University of Toronto, Toronto, ON M5S 3G4, Canada; E-Mails: huilin.hsu@mail.utoronto.ca (H.-L.H.); keith.leong@mail.utoronto.ca (K.R.L.); michael.halamicek@mail.utoronto.ca (M.H.); 2 Centre for Interdisciplinary Science, National Chiao Tung University, Hsinchu 30010, Taiwan; E-Mails: ijteng@nctu.edu.tw (I.J.T.); jyjuang@nctu.edu.tw (J.-Y.J.); 3 Department of Electrophysics, National Chiao Tung University, Hsinchu 30010, Taiwan; 4 Department of Materials Science and Engineering, I-Shou University, Kaohsiung 84001, Taiwan; E-Mail: srjian@gmail.com; 5 Department of Materials Science and Engineering, University of Toronto, Toronto, ON M5S 3E4, Canada

**Keywords:** RF-PEMOCVD, erbium metal-organic compound, deuterated amorphous carbon (a-C:D), fluorination

## Abstract

The integration of photonic materials into CMOS processing involves the use of new materials. A simple one-step metal-organic radio frequency plasma enhanced chemical vapor deposition system (RF-PEMOCVD) was deployed to grow erbium-doped amorphous carbon thin films (a-C:(Er)) on Si substrates at low temperatures (<200 °C). A partially fluorinated metal-organic compound, tris(6,6,7,7,8,8,8-heptafluoro-2,2-dimethyl-3,5-octanedionate) Erbium(+III) or abbreviated Er(fod)_3_, was incorporated *in situ* into a-C based host. Six-fold enhancement of Er room-temperature photoluminescence at 1.54 μm was demonstrated by deuteration of the a-C host. Furthermore, the effect of RF power and substrate temperature on the photoluminescence of a-C:D(Er) films was investigated and analyzed in terms of the film structure. Photoluminescence signal increases with increasing RF power, which is the result of an increase in [O]/[Er] ratio and the respective erbium-oxygen coordination number. Moreover, photoluminescence intensity decreases with increasing substrate temperature, which is attributed to an increased desorption rate or a lower sticking coefficient of the fluorinated fragments during film growth and hence [Er] decreases. In addition, it is observed that Er concentration quenching begins at ~2.2 at% and continues to increase until 5.5 at% in the studied a-C:D(Er) matrix. This technique provides the capability of doping Er in a vertically uniform profile.

## 1. Introduction

Incorporating optical technologies into microelectronic devices has been researched as a viable solution to overcome the speed bottlenecks associated with the ever shrinking device features [1]. The optical interconnect is able to transfer and process data at rates that are orders of magnitude higher than traditional electronic technologies, both within a Si chip and in chip-to-chip communications. In order to completely avail optical technologies, it is imperative to develop silicon compatible materials that enable light generation, guiding, switching, detection, modulation and amplification. To realize the co-existence of electrical and optical functions on the same Si chip platform, it is essential to develop Si compatible photonic materials. Further, the materials processing temperatures being below 400 °C is highly desirable [2] as this would meet the Si back end-of-line (BEOL) requirements in the current Si integrated circuit (IC) fabrication technology.

Crystalline silicon is not readily amenable for light emission due to its inherent indirect bandgap characteristic. Although, major breakthroughs in this field include the observation of an optical gain in Si nanocrystals [3], development of a Si Raman laser [4], electroluminescence for a Si diode [5], Si nanocrystal field-effect-transistors [6], and realization of a high-speed Si electro-optic modulator [7], these approaches have efficiency limitations. As well, the materials or the processing methods proposed require considerable development to ensure compatibility with current Si technology.

Ion implantation of light-emitting impurities, such as erbium (Er), into a variety of silica-based [8,9], ceramic [9], and Si-based [9,10] thin film hosts, has been a leading technique availed by the scientific community to efficiently produce photons from Si. The advantage of this approach is that standard Si technology can be deployed to introduce Er as a dopant. In addition, Er^3+^ ions can emit photons at 1.5 μm, which is a strategic wavelength for telecommunications due to a minimum in the absorption of silica fibers. However, high processing temperatures are required to grow silica-based (SiO_2_, phosphosilicate, borosilicate) and ceramic (Al_2_O_3_, Y_2_O_3_, LiNbO_3_) thin film hosts. These are incompatible with Si BEOL fabrication processes. Also, high temperature (>700 °C) post-deposition annealing processing is necessary to eliminate the ion implantation-induced damage, optically activate the Er^3+^ ions, and enhance the photoluminescence lifetime or quantum efficiency [9]. However, in many cases, high temperature annealing is not sufficient to remove the defects. For instance, ion implantation-induced defects still exist in high Er fluence implanted borosilicate glass. Despite subjecting the samples to a post-deposition annealing step, additional quenching sites coupled to O–H bonds are created. For crystalline Si based hosts, photoluminescence is severely quenched at room temperature as a result of the strong non-radiative processes that compete with the radiative Er de-excitation. Co-implantation of additional O atoms (highly preferred) reduces the Er segregation/precipitation by forming Er–O complexes, and increases the fraction of active Er^3+^ ions. To overcome the quenching issue, a SiO_2_ matrix containing Er-doped Si nanocrystals has been applied. The Si nanocrystals act as efficient sensitizers, which is attributed to an effective Er excitation cross section that is more than two orders of magnitude larger compared to the Er resonant absorption of a photon [11]. However, high temperature post-deposition annealing processing is inevitable in order to form Si nanocrystals inside a SiO_2_ matrix. High Er concentration and lower temperature quenching can be achieved in hydrogenated amorphous Si (a-Si:H) and/or porous Si. This is achieved by using low temperature processing methods, such as low pressure chemical vapor deposition (LPCVD) or plasma enhanced chemical vapor deposition (PECVD), both of which are Si BEOL compatible. Nevertheless, high temperature post-deposition annealing is required in order to observe photoluminescence in Er doped a-S:H and/or porous Si films.

Although extensive research on Er-implanted silicon and silica-based materials has been performed, Er doping in an amorphous carbon based host has received little attention [12–16]. Hydrogenated amorphous carbon (a-C:H) films, for example grown by a low-temperature PECVD method, exhibits compatible processing parameters with current CMOS fabrication technology. This facilitates integration and allows for reproducible, low-cost films. In addition, a-C based films possess a number of outstanding properties such as high chemical resistance, mechanical hardness, biocompatibility [17], and transparency in the infrared [18,19]. Due to their excellent tribiological properties, a-C:H films are widely used as protective coatings for hard disks and magnetic media, machine parts, optical windows and fibers, and other surfaces [20]. The specific properties of a-C:H films can be tailored over a wide range by adjusting the amount of sp^3^ and sp^2^ hybridized carbon, and the incorporated hydrogen content in the film via various deposition parameters and methods [19]. Moreover, these films can be deposited uniformly over a large area. These unique and versatile properties provide an impetus to utilize PECVD a-C films for specific optoelectronic applications.

The first reported demonstration of room-temperature photoluminescence (PL) from Er at 1.54 μm in a-C:H(Er) thin films was published in 2002 [12]. a-C:H(Er) films were deposited by magnetron sputtering of a graphite target that was partially covered by Er platelets in an Ar/C_6_H_12_ atmosphere. The Er concentration in the a-C:H(Er) films could be changed from 0.15 at% to1.2 at%. However, the PL intensity was relatively low. This was caused by the non-radiative relaxation pathway induced by C–H vibrations and the low optical band gap (~0.5 eV) of the sample [15]. In this deposition technique, the Er concentration depends highly on the degree of magnetron sputtering of the Er/graphite target. Accordingly, high Ar ion energy and flux are required to achieve high Er concentration. However, this causes a high concentration of *sp^2^* carbon and a low optical bandgap. The incorporation of an Er metal-organic compound into a carbon layer by the radio frequency PECVD method was demonstrated by Prajzlera *et al.* [14] in 2003, however, no PL spectra were presented. In 2009, Tsai *et al.* [16] grew a-C:H(Er) and a-C:D(Er) films via *in situ* thermal evaporation of the tris(2,2,6,6-tetramethyl-3-5 heptanedionato) erbium(+III), or Er(tmhd)_3_, compound in a DC saddle-field PECVD system. A PL signal was observed from both a-C:H(Er) and a-C:D(Er) films. However, the relative PL intensity from a-C:H(Er) was at least 10 times lower than from a-C:D(Er), attributed to the optical quenching from the highly abundant C–H bonds. Further, the Er(tmhd)_3_ metal-organic compound contains a high percentage of C–H bonds, 58.76 at%. Hence, this precursor is inherently inefficient at promoting Er^3+^ photoluminescence.

In this work, the feasibility of *in situ* growth of Er-doped a-C based thin films (a-C(Er)) at low temperatures (<200 °C) by simple occlusion of a metal-organic in a radio frequency plasma enhanced chemical vapor deposition (RF-PEMOCVD) system is investigated. The properties of the a-C host film and the incorporated Er concentration are independently controlled. The effect of RF power and substrate temperature on the photoluminescence of a-C(Er) films is investigated. The film structure, incorporated Er concentration, and the effect of the change in optical properties of host a-C are also discussed.

## 2. Results and Discussion

### 2.1. Photoluminescence Enhancement by Deuteration of a-C host

**Figure 1.**
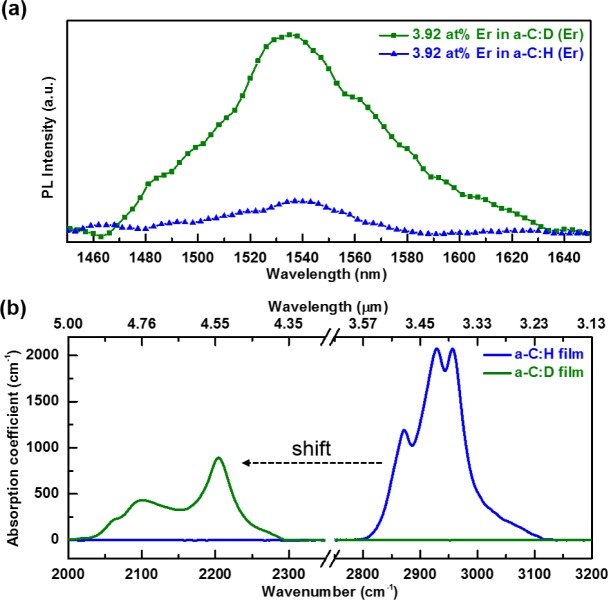
(**a**) Comparison of PL intensity of a-C:D(Er: 3.92 at% ) and a-C:H(Er: 3.92 at %) films prepared using RF power of 40 W, precursor gas flow rate of 40 sccm, deposition pressure of 120 mTorr, substrate temperature of 80 °C, and Er(fod)_3_ powder evaporation temperature of 150 °C; (**b**) Comparison of FTIR spectra of the a-C:D and a-C:H host films free of Er metal-organic prepared under otherwise identical deposition conditions.

In the Er metal-organic compound, non-radiative deactivation channels exist near the active Er^3+^ ions due to the surrounding ligands. Any additional C–H and O–H bonds from the host material would cause additional quenching of the Er^3+^ luminescence. Thus, it is not surprising that there are only a few publications [12–16] which demonstrate the photoluminescence of Er complexes in hydrogenated amorphous carbon (a-C:H), or other carbon-based related materials such as polymers [21,22]. To suppress the quenching effect from the Er(fod)_3_ ligands and the host material, the precursor gas used to deposit the host a-C was changed from methane (CH_4_) to deuterated methane (CD_4_). The resultant PL spectrum is depicted in Figure 1a. The room temperature PL spectra centered at 1540 nm corresponds to the ^4^*I*_13/2_ to ^4^*I*_15/2_ electronic transition of Er^3+^ ions. The spectral width of the emission band is due to inhomogeneous and homogeneous broadening in addition to Stark splitting of the Er^3+^ excited and ground states. The PL peak is wider than those of other Er-implanted silicate glasses [9], indicating that Er^3+^ possesses a variety of local bonding environments in the a-C matrix. Its 65 nm full width at half-maximum (FWHM) suggests the potential of enabling a wide gain band width for optical amplification. It is observed that the intensity of PL is enhanced by a factor of six with the same Er concentration in the a-C:D matrix. This confirms that Er luminescence is enhanced through the substitution of H with D in the a-C host samples prepared by the RF-PEMOCVD method.

**Figure 2.**
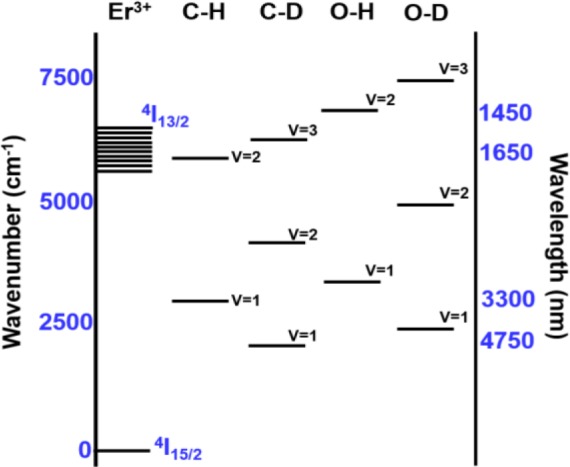
Illustration of energy levels of vibrational modes in hydrogen and deuterium containing organic media. Reprinted/Reproduced with permission from [23]. American Institute of Physics 1974 AIP Publishing.

The enhancement of PL is a result of the weaker interaction strength between Er^3+^ and the C–D and O–D third harmonic vibrations, compared to the interaction strength between Er^3+^ and the C–H and O–H second harmonic vibrations. The C–H and O–H second harmonic vibrations (5900 and 6900 cm^−1^, respectively) approximately match (~1500 nm) the radiative transition from the first excited state ^4^*I*_13/2_ to the ground state ^4^*I*_15/2_ in Er^3+^ ions (~6500 cm^−1^) [23] as seen in Figure 2. Accordingly, if excited Er^3+^ ions are disturbed nearby C–H and O–H oscillators, a non-radiative transition transpires. By applying the undistorted oscillator model [24], the transition probability between Er^3+^ and the vibrational modes of the host a-C:D significantly decreases. Furthermore, by directly comparing the FTIR spectra of the host a-C:H and a-C:D films, shown in Figure 1b, the impact of the host a-C materials on Er^3+^ PL quenching can be clearly seen. Deuteration of the host a-C effectively shifts the absorption of the first vibrational mode (υ = 1) to lower wavenumber (longer wavelength) as seen in Figure 2. Also, the magnitude of the absorption has decreased by at least 50% as indicated in Figure 1b. Moreover, the higher harmonic vibrational modes are much weaker, implying much lower absorption of C–D*x* in the third harmonic (υ = 3) than that of C–H*x* in the second harmonic (υ = 2). Thus, this leads to an increase in the efficiency of the PL at 1540 nm for a-C:D (Er) films.

### 2.2. Effe**c**t of RF Power and Substrate Temperature on a-C:D(Er) Film

Two key parameters that are found to influence the concentration of Er occluded in the a-C:D(Er) film are the RF power and substrate temperature. Table 1 lists the ratios of the atomic concentrations and the relative (and absolute) atomic concentrations of the relevant elements in the as-received stoichiometric Er(fod)_3_ compound, thermally evaporated (abbreviated TE) Er(fod)_3_, a-C:H(Er), and in the seven a-C:D(Er) films deposited under varying conditions as determined from XPS measurements. In comparing the a-C:D(Er) film deposited at an RF power of 40 W to the stoichiometric Er(fod)_3_ compound, a few results are observed. The [O]/[C] ratios are very similar, ~0.19 in the a-C:D(Er) film compared to ~0.20 in the stoichiometric Er(fod)_3_. Meanwhile, the [O]/[Er] and [C]/[Er] ratios are approximately 4.5× smaller in the film, while the [F]/[Er] ratio is 2.3× smaller in the film. This suggests that Er incorporation is being promoted in the film. Moreover, since the [F]/[C] ratio is 2× larger in the film, fluorine incorporation is also being promoted. Clearly, the plasma environment causes significant dissociation of the Er(fod)_3_ compound. The loss of C and O atoms is attributed to the loss of large C*_x_*O*_y_* (or deuterated C*_x_*O*_y_*) fragments. The increase in the F concentration is due to the selective incorporation of C*_m_*F*_n_* fragments.

**Figure 3.**
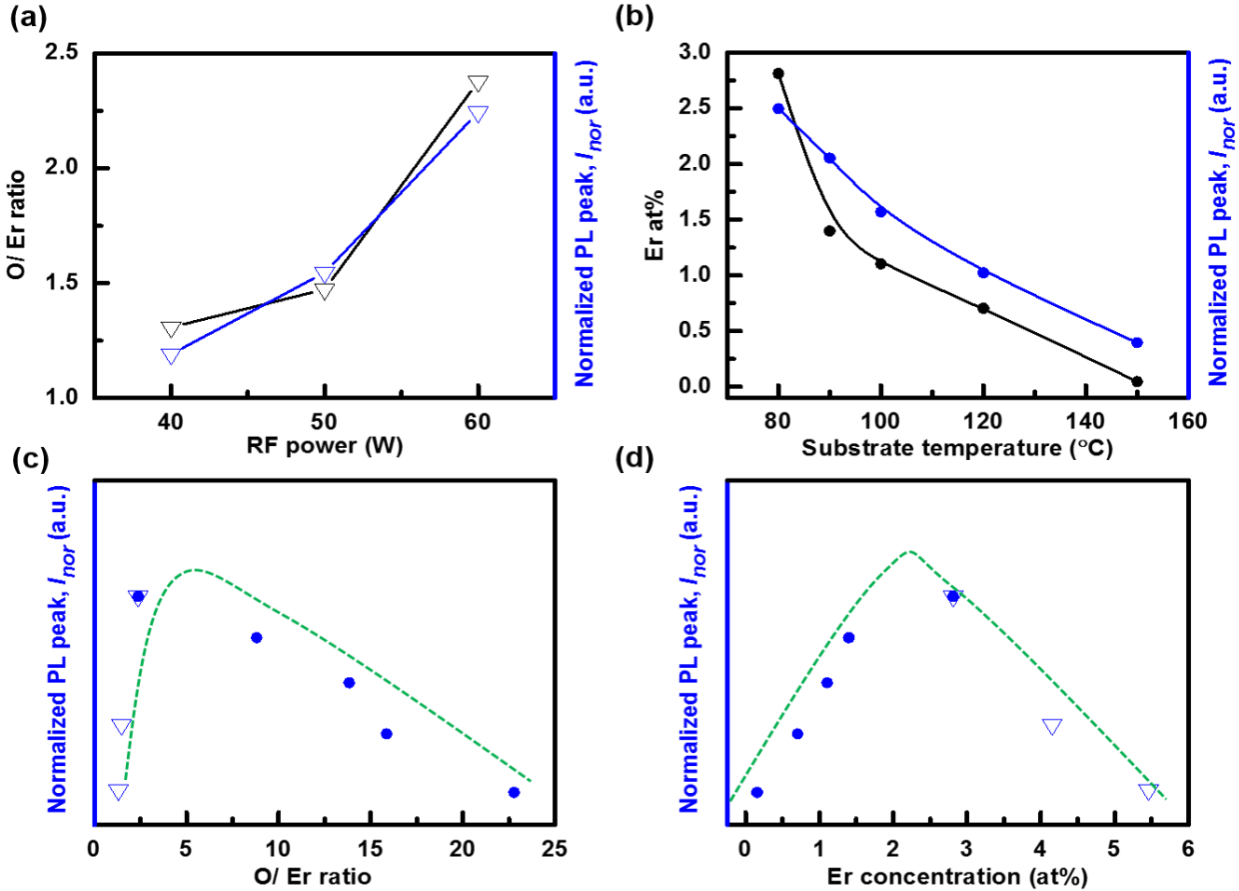
(**a**) The [O]/[Er] ratio (black triangle) and normalized PL peak intensity (blue triangle) as a function of the applied RF power with a substrate temperature of 80 °C; (**b**) The Er concentration (solid black circle) and normalized PL peak intensity (solid blue circle) as a function of the substrate temperature with an RF power of 60 W. The normalized PL peak intensity is shown to depend critically on the (**c**) [O]/[Er] ratio; and (**d**) [Er]. The lines are guides to the eye.

The Er concentration in a-C:D(Er) decreases with increasing RF power as shown in Table 1. This is evident from the increasing [O]/[Er], [F]/[Er], and [C]/[Er] ratios. The [C] increases with increasing RF power. This is observed through the increase in the [C]/[Er] ratio and decrease in the [O]/[C] and [F]/[C] ratios. The [O] increases when compared to the [F] with increasing RF power, as seen through the increase in the [O]/[F] ratio. The decrease in the relative Er, F, and O concentrations relative to [C] with increasing RF power is due to the increase in the plasma density. As the RF power increases, the dissociation of the precursors (CD_4_ and Er(fod)_3_) increases. Thus, more C is being incorporated via CD_4_. The relative [F] is still quite large, 29 at% for the 60 W sample, which is considered to aid the PL efficiency by further reducing the C–H quenching as discussed below in the Section 3.2. Also, note the decrease in the relative [Er], from 5.5 at% at 40 W to 2.8 at% at 60 W. Despite this decrease, the [O]/[Er] ratio increases from 1.31 to 2.38 as observed in Table 1. The increase in [O]/[Er] ratio is considered to increase the number of erbium-oxygen bonded complexes, which are responsible for the increase in the PL signal with increasing RF power as seen in Figure 3a.

Another means of controlling the a-C:D(Er) film structure, and hence the PL efficiency, is through the temperature of the growth surface. The Er(fod)_3_ evaporation temperature is 150 °C. Hence, the temperature of the substrate will affect the relative sticking coefficient of the Er metal-organic and its associated molecular fragments. Analyzing the XPS spectra for the films grown as the substrate temperature was varied from 80 to 150 °C yields a number of results. The [F] decreases rapidly with increasing substrate temperature. This is seen through the increasing [O]/[F] ratio and the decreasing [F]/[Er] and [F]/[C] ratios. The [Er] is also decreasing with increasing substrate temperature as shown in Figure 3b. This is evident from the increase in the [O]/[Er] and [C]/[Er] ratios. In examining the [O]/[C] ratio, the [O] increases, peaks at a substrate temperature of about 100 °C, then decreases as the substrate temperature increases. The considerable loss of F is associated with a drop in the deposition of fluorinated Er(fod)_3_ molecular fragments. This is presumably due to either an increased desorption rate or a lower sticking coefficient of the fluorinated fragments. This is supported by the rise in the [O]/[C] ratio, which implies that oxygenated molecular fragments (C*_x_*O*_y_*) are preferentially incorporated into the film. The boiling temperatures of C*_x_*O*_y_* molecules are typically higher than C*_m_*F*_n_* molecules. Moreover, it is well known that the deposition rate of a-C:H decreases with increasing substrate temperature. Hence, the increase in the [C] is not necessarily due to CD_4_ species. Despite the relatively large decrease in the [F], and the smaller decrease in the [Er], the relative PL intensity slowly decreases as indicated in Figure 3b. This is attributed to the increasing [O]/[Er] ratio as seen in Figure 3c. Hence, more Er atoms are considered to be in the 3+ state. As well, the loss of a large fraction of the fluorinated ligands which in turn have likely been replaced by the deuterated film is considered to suppress the optical quenching of excited Er^3+^ ions. As the substrate temperature is increased beyond 100 °C, there is an increase in the desorption rate or a lowering of the sticking coefficient of O containing species. At a substrate temperature of 150 °C, the majority of the film is deuterated amorphous carbon with minute O, F, and Er concentration. The [O]/[Er] ratio is 22.78 and a PL signal is present, although the peak is approximately 7× lower than the maximum PL signal due to the lower Er concentration. Examining the change in the optical properties of a-C:D host, it is observed that *k* increases from 1.65 × 10^−3^ to 3.2 × 10^−3^ and *E*_04_ decreases from 3.81 to 3.41 eV as the substrate temperature is increased from 80 to 150 °C. This minute change is not expected to significantly influence the optical pumping, absorption and PL.

### 2.3. Er Quenching Concentration in a-C:D(Er) Film

In order to study the concentration dependence of the incorporated Er(fod)_3_ on luminescence of the a-C:D matrix in more detail, the PL intensity which peaks at 1540 nm is normalized to the film thickness, denoted *I*_nor_. Figure 3d plots the *I*_nor_ as a function of the Er concentration, *N*_Er_. *I*_nor_ is proportional to σφ*N*Γ/Γ_dar_ under the continuous laser pumping condition [25], where σ is the excitation cross section, φ is the photon flux, *N* is the optically active Er concentration, Γ is the lifetime, and Γ_dar_ is the radiative lifetime. The increase in *N*_Er_ will result in a linear increase in *I*_nor_ if all the incorporated Er atoms are optically active, *i.e.*, *N~N*_Er_, and if there are no quenching effects to adversely affect σ and Γ/Γ_dar_. In Figure 3d, it is observed that *I*_nor_ increases linearly up to an Er concentration of approximately 2.2 at% and thereafter begins to drop. The latter suggests a reduction in lifetime Γ as *N*_Er_ increases beyond this point. It indicates that the Er concentration quenching effect sets in after the Er concentration reaches ~2.8 at% and continues to increase until 5.5 at% at which point the PL becomes very weak.

### 2.4. Er Oxidation State in a-C:D(Er) Film

**Figure 4.**
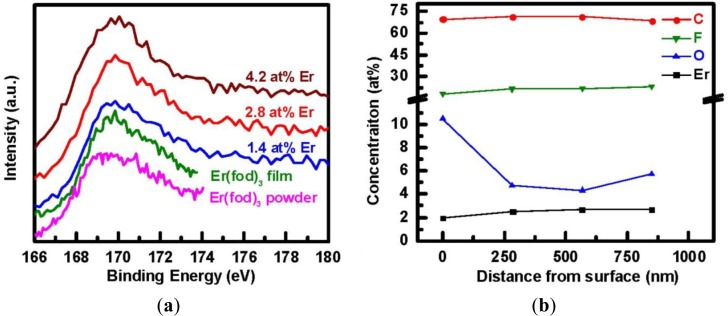
(**a**) XPS spectra of the three a-C:D(Er) films, Er(fod)_3_ film (evaporated in the vacuum chamber with CD_4_ precursor gas flowing without plasma ignition), and the as-received stoichiometric Er(fod)_3_ powder. The curves have been shifted vertically for clarity of presentation; (**b**) Depth profile of C, F, O, and Er concentrations in a-C:D(Er) film deposited at 60 W of RF power and 80 °C as determined from XPS measurements.

The Er4d XPS spectra of the Er(fod)_3_ powder, the evaporated Er(fod)_3_ film, and the three a-C:D(Er) samples with different Er concentrations are compared in Figure 4a. The spectra reveal similar spectral characteristic feature at a binding energy of approximately 169.5 eV for all of the samples. This is attributed to the 4d levels in the Er^3+^ ions forming a multiplet through the interaction with the unfilled shell. Hence, the incorporated Er in a-C:D(Er) films is similar to the Er in the Er(fod)_3_ compound. Moreover, it suggests partial preservation of the Er^3+^ state in a-C:D(Er) films. The presence of a PL signal also supports this result, in spite of the fact that the O/Er ratio is less than 6 as is the case in the pristine Er(fod)_3_ powder. The depth distribution as depicted in Figure 4b reveals a uniform concentration of Er throughout the film of 850 nm in thickness. This is in contrast to ion-implantation of Er where the optically active ions are always located near the surface [9]. It is noteworthy that the high oxygen concentration at the surface is simply surface contamination. Optimization of process parameters and the effect of F composition on optical properties of the carbon matrix, as well as understanding the electronic transfer mechanism are currently under investigation.

Currently deuterated methane (CD_4_) gas is more expensive than standard methane (CH_4_) gas. Thus, a path towards device manufacturing with deuteration may include schemes such as the mixing of CH_4_ and CD_4_ or CH_4_ and D_2_ as the precursor gas. Clearly, optimization of the device processing would be required in order to maintain the PL enhancement. Furthermore, even though the deployment of CD_4_ leads to PL enhancement in the current deposition method, the ligand of the Er metal-organic compound is in a hydrogenated form. This contrasts the extensive research of PL enhancement by deuterated ligands and/or deuterated polymer host [26,27], where the synthesis procedures are more complicated and could result in higher manufacturing costs.

## 3. Experimental Section

### 3.1. RF-PEMOCVD and Sample Preparation

**Figure 5.**
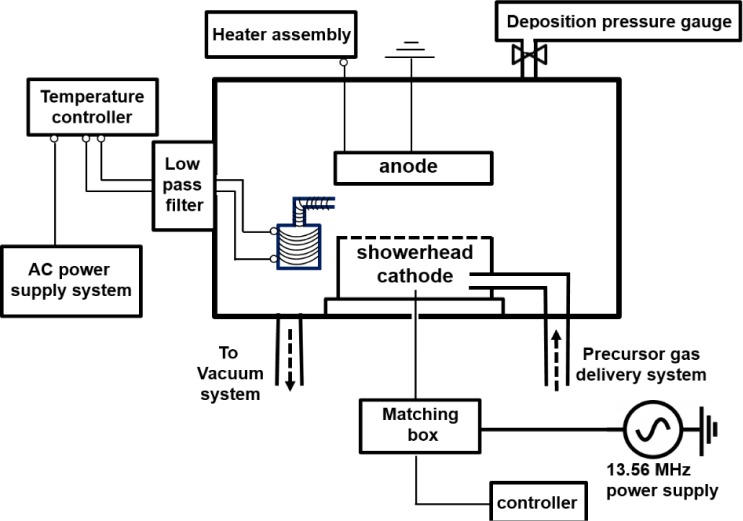
Schematic diagram of the RF-PEMOCVD system used for the preparation of a-C:D(Er) films.

A capacitively coupled RF-PEMOCVD system, shown in Figure 5, with a base pressure of 5 × 10^−5^ Torr was deployed to deposit Er metal-organic doped a-C thin films. An ac-powered thermal evaporator situated next to the RF-powered electrode (cathode), inside the deposition chamber was utilized to *in situ* dope the Er metal-organic compound concurrent with the a-C film deposition. A thermocouple is embedded on the bottom of the evaporator, providing feedback to the temperature controller for precise control of the evaporation temperature. A low pass filter is connected using Cu conductors between the thermal evaporator inside the chamber and the external ac power supply in order to prevent the RF plasma power source from dissipating into the temperature controller or the ac power line. The temperature of the vapor delivery nozzle was kept higher than that of the bottom of the container, by 30–50 °C, so as to avoid condensation of the Er metal-organic vapor on the delivery nozzle. The *in situ* thermal evaporation technique provides the potential of doping Er in a vertically uniform profile as well as design specific concentration profiles. This is unlike the ion-implantation technique, post solution immersion method, or vapor exposure to an Er source procedure [14] where the optically active ions are invariably located near the surface. Further, film damage caused by the high energy ions in the ion-implantation process is avoided. Moreover, the present approach does not require an additional sacrificial metal layer which is typical in the implantation process for non-metallic thin film hosts so as to prevent electrical charging. Also, compared to the co-sputtering [10] or pulsed laser ablation [13,28] of an Er-containing target, this method enables independent control of the Er concentration profile and optical properties of the host material.

For a-C(Er) samples, the hydrocarbon gas flow rate was 20 sccm and the chamber pressure was 60 mTorr unless otherwise stated. For the films where the RF power was varied, the power was varied from 40 to 60 W while the substrate temperature was kept constant at 80 °C. For the films where the substrate temperature was varied, the temperature was varied from 80 to 150 °C while the RF power was kept constant at 60 W. Double-side polished crystalline silicon substrates with resistivity of 30 ohm-cm were subjected to the standard CMOS cleaning procedure before being loaded into the chamber.

### 3.2. Incorporated Er Metal-organic Compound

**Figure 6.**
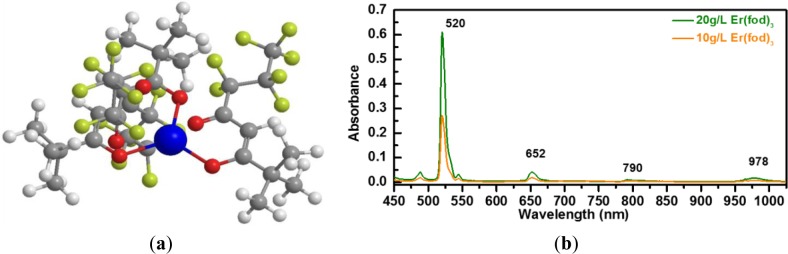
(**a**) Illustration of the Er metal-organic compound, Er(fod)_3_, with chemical structure Er(C_10_H_10_O_2_F_7_)_3_. The large center blue atom represents Er, red atoms represent O, dark grey atoms represent C, yellow atoms represent F, and white atoms represent H; (**b**) The absorption spectra of Er(fod)_3_ metal-organic compound dissolved in d-chloroform solvent.

The Er metal-organic compound tris (6,6,7,7,8,8,8-heptafluoro-2,2-dimethyl-3,5-octanedionate) Erbium(+III), abbreviated (Er(fod)_3_), having the chemical structure Er (C_10_H_10_F_7_O_2_)_3_ and illustrated in Figure 6a, is selected as the doping candidate for a-C(Er) films. It is presumed that the optically active Er^3+^ ions originating from the Er(fod)_3_ compound can be preserved under appropriate deposition conditions, similar to previous demonstration of other Er metal-organic compound such as Er(thmd)_3_ [29,30]. Accordingly, these Er^3+^ ions would not require a high temperature post-deposition annealing step to be optically activated and/or to repair film damage. In a similar compound, tris(2,2,6,6-tetramethyl-3-5 heptanedionato) erbium(+III) powder, denoted Er(tmhd)_3_, Er is coordinated to six oxygen atoms. It has been demonstrated that the Er local environment in an as-deposited a-Si:H(Er) sample prepared by PECVD is very similar to Er_2_O_3_. This bonding environment has efficiently promoted the optically emitting centers [30]. In this work, a controlled vapor flux of Er(fod)_3_ compound is introduced by thermal evaporation at 150 °C. The vapor flux is mixed with the hydrocarbon plasma for a-C(Er) film formation. In order to determine the proper wavelength of the pumping source for the Er(fod)_3_ compound, the absorption spectrum was collected with an UV-Vis-NIR spectrometer (PerkinElmer, Waltham, MA, USA). As displayed in Figure 6b, the absorption spectrum for the Er(fod)_3_ compound dissolved in a d-chloroform solvent suggests that the potential excitation source can be near 520 nm.

Another advantage of the Er(fod)_3_ compound deployed in this work is the partial fluorination of the hydrogen-containing ligands which is expected to reduce the C–H quenching effect. The ligands and coordinated solvent molecules of Er metal-organic compounds usually contain C–H and O–H bonds. Accordingly, if excited Er^3+^ ions are disturbed nearby C–H and O–H oscillators, a non-radiative transition occurs [23]. This would dramatically reduce the luminescence efficiency. It has also been quantitatively demonstrated that a three order of magnitude increase in the radiative lifetime can be achieved by increasing the distance between the neighboring C–H and O–H bonds in the ligands and the Er^3+^ ion [31]. However, the presence of C–H vibrational oscillators within a sphere of at least 20 Å from the Er^3+^ ion can still be an effective de-excitation site [31]. Thus, fluorination of the hydrogen-containing ligands is expected to reduce the non-radiative deactivation channels due to C–H bonds. This will enable an enhancement in the Er^3+^ luminescence efficiency [27,32].

### 3.3. Film Characterization

#### 3.3.1. Photoluminescence

Photoluminescence spectra of Er metal-organic doped a-C based films were collected at room temperature to verify the optical activity of Er in the a-C films. A continuous wave diode-pumped solid-state 532 nm laser with a power density of 80 mW/mm^2^ was used as the excitation source, in keeping with the observed absorption peak for the Er(fod)_3_ shown in Figure 6b. A thermoelectrically cooled InGaAs photodiode, 800–1700 nm detection range, with standard lock-in techniques was employed. A single pass monochromator was utilized to disperse the emitted PL. The energy of the laser is near resonance with the ^4^*S*_3/2_ excited level of Er ions. The excited Er ions decay to ^4^*I*_13/2_ level through the fast non-radiative transition and then emit at 1540 nm through ^4^*I*_13/2_ to ^4^*I*_15/2_ transition.

#### 3.3.2. X-Ray Photoelectron Spectroscopy

X-ray photoelectron spectroscopy (XPS) was employed to quantitatively characterize the elemental composition and the depth distribution of a-C(Er) films. The XPS spectra were collected from the surface of the sample using a monochromatic Al Kα X-ray source in a Thermo Scientific K-Alpha spectrometer (ThermoFisher Scientific, Waltham, MA, USA) with an ultrahigh vacuum of the order of 10^−9^ Torr. The samples were attached to a stainless steel holder using Cu conductive double-sided tape. The top of the sample was also grounded by Cu tape in order to prevent severe charging effects.

#### 3.3.3. Spectroscopic Ellipsometry and Fourier Transform InfraRed Spectroscopy

The extinction coefficient *k* and optical bandgap *E*_04_ of the host deuterated amorphous carbon (a-C:D) films were probed through spectroscopic ellipsometry. The measurements were carried out using an UV-Vis-NIR spectroscopic ellipsometer (Sopralab, Paris, France). The wavelength range was 350–1700 nm at an incident angle of 75°. The spectra were analyzed by regression fitting using the linear Levenberg-Marquard algorithm method with a maximum of 1000 iterations using a three-layer optical structure comprising void (ambient)/a-C layer/c-Si substrate. A first-order initial thickness approximation of the a-C film was estimated from profilometry measurements. The five constants of Forouhi-Bloomer dispersion model [33] and thickness of the a-C:D layer were allowed to vary during the fitting process. The optical bandgap *E*_04_, defined as the photon energy at which the absorption coefficient α(=4π*k*/λ) is equal to 10^4^ cm^−1^, was determined from the extinction coefficient *k*; λ is the wavelength. The fits yielded a coefficient of regression R^2^~0.99 and the error of the six fitting parameters was less than +/−10%, indicating the model was appropriate for the a-C:D films.

The absorption spectra of the host a-C films were characterized by Fourier Transform InfraRed (FTIR) Spectroscopy using a Perkin Elmer 2000 spectrometer (PerkinElmer, Waltham, MA, USA) with a resolution of 4 cm^−1^. The transmission spectra were background corrected for the interference patterns emerging due to multiple reflections in the film. The absorption was determined using the following relation:

$$\alpha (\upsilon )=-\frac{1}{d}\text{ln}T$$ (1)

where *α*(*υ*) is the absorption constant, *d* is the thickness of the film, and *T* is the normalized transmission of the a-C film with the background removed.

## 4. Conclusions

The feasibility of the *in situ* growth of Er-doped a-C thin films on Si substrates at low temperature (<200 °C) by a simple one-step metal-organic radio frequency plasma-enhanced chemical vapor deposition system was successfully demonstrated. By adopting a new Er metal-organic precursor with partial fluorination, Er(fod)_3_, the optically active Er^3+^ ions are conserved, thus avoiding a subsequent high temperature annealing procedure and accordingly resulting in room temperature luminescence. Furthermore, the enhancement of the PL was demonstrated via deuteration of the a-C host by effectively shifting the quenching vibrational modes to lower wavenumbers and decreasing the respective magnitudes of absorption. The effect of RF power and substrate temperature on the Er concentration, [O]/[Er] ratio, and the respective PL intensity of the a-C:D(Er) film was investigated. It was observed that the PL signal increases with increasing RF power, which is attributed to an increase in [O]/[Er] ratio and hence the erbium-oxygen coordination number. The relatively large [F] is deemed to contribute to the enhancement in PL efficiency. In addition, PL intensity and [Er] decrease with increasing substrate temperature; the decrease in [Er] is attributed to an increased desorption rate or a lower sticking coefficient of the fluorinated fragments during film growth. By examining the relationship of the normalized PL intensity, *I*_nor_, as a function of the Er concentration, it is observed that Er concentration quenching begins at ~2.2 at% and continues to increase until 5.5 at% at which point the PL is very weak. Furthermore, the foregoing indicate the preservation of the Er^3+^ state in a-C:D(Er) films was achieved. The *in situ* thermal evaporation technique provides the capability of doping Er in a uniform profile vertically/depth-wise.

## Figures and Tables

**Table 1.** Ratios of atomic concentrations and relative/absolute atomic concentrations of relevant elements in as-received stoichiometric Er(fod)_3_ compound, thermally evaporated (TE) Er(fod)_3_, and in seven a-C:D(Er) films deposited under varying conditions as determined from XPS measurements.

Sample	RF Power (W)	Substrate Temperature (°C)	C at%	Er at%	F at%	O at%	[O]/[Er]	[F]/[Er]	[C]/[Er]	[O]/[C]	[F]/[C]	[O]/[F]
Er(fod)_3_	–	–	34.1	1.1	23.9	6.8	6.00	21.00	30.00	0.20	0.70	0.29
TE Er(fod)_3_	–	–	71.3	1.0	21.5	6.3	6.33	21.74	72.08	0.09	0.30	0.29
a-C:H(Er)	40	80	54.6	3.9	35.4	6.0	1.54	9.08	14.00	0.11	0.65	0.17
1	40	80	36.9	5.5	50.5	7.1	1.31	9.26	6.75	0.19	1.37	0.14
2	50	80	52.3	4.2	37.4	6.1	1.47	9.02	12.60	0.12	0.72	0.16
3	60	80	61.5	2.8	29.0	6.7	2.38	10.33	21.88	0.11	0.47	0.23
4	60	90	77.9	1.4	5.4	12.3	8.81	3.85	55.66	0.16	0.07	2.29
5	60	100	79.6	1.1	4.0	15.3	13.83	3.60	72.14	0.19	0.05	3.84
6	60	120	85.7	0.7	2.4	11.2	15.86	3.46	121.58	0.13	0.03	4.58
7	60	150	95.9	0.2	0.4	3.5	22.78	2.37	618.45	0.04	0.00	9.61

## References

[1] Paniccia M. (2010). Integrating silicon photonics. Nat. Photon.

[2] Xiao H. (2012). Introduction to Semiconductor Manufacturing Technology.

[3] Pavesi L., Dal Negro L., Mazzoleni C., Franzo G., Priolo F. (2000). Optical gain in silicon nanocrystals. Nature.

[4] Rong H., Liu A., Jones R., Cohen O., Hak D., Nicolaescu R., Fang A., Paniccia M. (2005). An all-silicon Raman laser. Nature.

[5] Green M.A., Zhao J., Wang A., Reece P.J., Gal M. (2001). Efficient silicon light-emitting diodes. Nature.

[6] Walters R.J., Bourianoff G.I., Atwater H.A. (2005). Field-effect electroluminescence in silicon nanocrystals. Nat. Mater.

[7] Xu Q., Schmidt B., Pradhan S., Lipson M. (2005). Micrometre-scale silicon electro-optic modulator. Nature.

[8] Miritello M., Lo Savio R., Iacona F., Franzò G., Irrera A., Piro A.M., Bongiorno C., Priolo F. (2007). Efficient luminescence and energy transfer in erbium silicate thin films. Adv. Mater.

[9] Polman A. (1997). Erbium implanted thin film photonic materials. J. Appl. Phys.

[10] Weiser G., Kühne H., Terukov I.E. (2004). Energy transfer to Er^3+^ ions in a-Si_1−_*_x_* C*_x_*:H alloys: Emission at 1.54 mm wavelength. Phys. Status Solidi C.

[11] Priolo F., Franzo G., Pacifici D., Vinciguerra V., Iacona F., Irrera A. (2006). Role of the energy transfer in the optical properties of undoped and Er-doped interacting Si nanocrystals. J. Appl. Phys.

[12] Baranov A.M., Sleptsov V.V., Nefedov A.A., Varfolomeev A.E., Fanchenko S.S., Calliari L., Speranza G., Ferrari M., Chiasera A. (2002). Erbium photoluminescence in hydrogenated amorphous carbon. Phys. Status Solidi B.

[13] Foong Y.M., Hsieh J., Li X., Chua D.H.C. (2009). The study on the effect of erbium on diamond-like carbon deposited by pulsed laser deposition technique. J. Appl. Phys.

[14] Prajzler V., Huttel I., Nekvindova P., Schrofel J., Mackova A., Gurovic J. (2003). Erbium doping into thin carbon optical layers. Thin Solid Films.

[15] Speranza G., Calliari L., Ferrari M., Chiasera A., Tran Ngoc K., Baranov A.M., Sleptsov V.V., Nefedov A.A., Varfolomeev A.E., Fanchenko S.S. (2004). Erbium-doped thin amorphous carbon films prepared by mixed CVD sputtering. Appl. Surf. Sci.

[16] Tsai R.Y.C., Qian L., Alizadeh H., Kherani N.P. (2009). Room-temperature photoluminescence in erbium-doped deuterated amorphous carbon prepared by low-temperature MO-PECVD. Opt. Express.

[17] McLaughlin J.A., Maguire P.D. (2008). Advances on the use of carbon based materials at the biological and surface interface for applications in medical implants. Diam. Relat. Mater.

[18] Patsalas P. (2011). Optical properties of amorphous carbons and their applications and perspectives in photonics. Thin Solid Films.

[19] Robertson J. (2002). Diamond-like amorphous carbon. Mater. Sci. Eng. R Rep.

[20] Piazza F., Grambole D., Schneider D., Casiraghi C., Ferrari A.C., Robertson J. (2005). Protective diamond-like carbon coatings for future optical storage disks. Diam. Relat. Mater.

[21] Wong W.H., Chan K.S., Pun E.Y.B. (2005). Ultraviolet direct printing of rare-earth-doped polymer waveguide amplifiers. Appl. Phys. Lett.

[22] Wong W.H., Pun E.Y.B., Chan K.S. (2004). Er^3+^–Yb^3+^ codoped polymeric optical waveguide amplifiers. Appl. Phys. Lett.

[23] Haas Y., Stein G., Wurzberg E. (1974). Radiationless transitions in solutions: Isotope and proximity effects on Dy^3+^ by C–H and C–N bonds. J. Chem. Phys.

[24] Siebrand W. (1967). Radiationless transitions in polyatomic molecules. I. Calculation of Franck-Condon Factors. J. Chem. Phys.

[25] Saleh B.E.A., Teich M.C. (2007). Fundamentals of Photonics.

[26] Hasegawa Y., Wada Y., Yanagida S. (2004). Strategies for the design of luminescent lanthanide(III) complexes and their photonic applications. J. Photochem. Photobiol. C.

[27] Kuriki K., Koike Y., Okamoto Y. (2002). Plastic optical fiber lasers and amplifiers containing lanthanide complexes. Chem. Rev.

[28] Foong Y.M., Hsieh J., Li X., Chua D.H.C. (2010). Comparative study between erbium and erbium oxide-doped diamondlike carbon films deposited by pulsed laser deposition technique. J. Vac. Sci. Technol. A.

[29] Terukov E.I., Konkov O.I., Kudoyarova V.K., Koughia K.V., Weiser G., Kühne H., Kleider J.P., Longeaud C., Brüggemann R. (2000). Erbium incorporation in plasma-deposited amorphous silicon. J. Noncryst. Solids.

[30] Cínthia Piamonteze L.R.T., Hélio Tolentino M.C.M.A., Gerhard W., Eugeny T. (2000). Er environment in a-Si:H(Er) prepared by PECVD. Mater. Res. Soc. Symp. Proc.

[31] Winkless L., Tan R.H.C., Zheng Y., Motevalli M., Wyatt P.B., Gillin W.P. (2006). Quenching of Er(III) luminescence by ligand C–H vibrations: Implications for the use of erbium complexes in telecommunications. Appl. Phys. Lett.

[32] Monguzzi A., Tubino R., Meinardi F., Biroli A.O., Pizzotti M., Demartin F., Quochi F., Cordella F., Loi M.A. (2008). Novel Er^3+^ perfluorinated complexes for broadband sensitized near infrared emission. Chem. Mater.

[33] Forouhi A.R., Bloomer I. (1986). Optical dispersion-relations for amorphous-semiconductors and amorphous dielectrics. Phys. Rev. B.

